# Treatment of Shiga-Toxin Hus with Severe Neurologic Features with Eculizumab

**DOI:** 10.1155/2021/8053246

**Published:** 2021-11-13

**Authors:** Jacob H. Umscheid, Collin Nevil, Rhythm Vasudeva, Mohammed Farhan Ali, Nisha Agasthya

**Affiliations:** ^1^University of Kansas, School of Medicine, Wichita, USA; ^2^Children's Mercy Hospital, Department of Nephrology, Kansas City, USA

## Abstract

Hemolytic Uremic Syndrome (HUS) is a constellation of microangiopathic hemolytic anemia, thrombocytopenia, and acute renal failure. Shiga toxin-producing *Escherichia coli*- (STEC-) mediated HUS is a common cause of acute renal failure in children and can rarely result in severe neurological complications such as encephalopathy, seizures, cerebrovascular accidents, and coma. Current literature supports use of eculizumab, a monoclonal antibody that blocks complement activation, in atypical HUS (aHUS). However, those with neurologic complications from STEC-HUS have complement activation and deposition of aggregates in microvasculature and may be treated with eculizumab. In this case report, we describe a 3-year-old boy with diarrhea-positive STEC-HUS who developed severe neurologic involvement in addition to acute renal failure requiring renal replacement therapy. He was initiated on eculizumab therapy, with clinical improvement and organ recovery. This case highlights systemic complications of STEC-HUS in a pediatric patient. The current literature is limited but has suggested a role for complement mediation in cases with severe complications. We review the importance of early recognition of complications, use of eculizumab, and current data available.

## 1. Introduction

Shiga toxin-producing *Escherichia coli* (STEC) is a well-known etiology for gastroenteritis and colitis; however, the toxin can traverse through the gastrointestinal system into the bloodstream causing injury to vascular endothelial cells [1, 2]. This results in a microangiopathic hemolytic anemia, thrombocytopenia, and renal failure, also known as hemolytic uremic syndrome (HUS) [1, 3]. HUS is a common cause of acute kidney injury in children, accounting for around 17% of cases and usually between the ages of 1 to 5 years (74%) [4]. This is secondary to toxin binding to the renal endothelial cells, but may extend to extrarenal tissue resulting in severe neurological complications of altered mental status, seizures, cerebrovascular accidents, and coma [5]. When neurologic complications of HUS are present, the risk of severe sequelae and mortality significantly increases [6, 7]. In a large retrospective cohort study of 3915 pediatric patients, 10.4% developed acute neurologic manifestations and had significantly worse mortality (13.9%) when compared to HUS patients without neurologic involvement (1.8%) [6].

Endothelial injury to small vessels through Shiga-like toxin binding to globotriaosylceramide (Gb3) receptors and microvascular thrombosis results in the majority of damage to organs in HUS [8]. The Gb3 receptor is present not only throughout renal and cerebral microvascular endothelial cells but also on neuronal cells, placing the entire central nervous system (CNS) at risk for injury [8]. Also seen is an increase in cytokine production in response to Shiga-like toxin at the blood-brain barrier resulting in cytotoxic effects to the CNS parenchyma [7, 9–11]. This increased inflammatory response may suggest why immunomodulators, such as eculizumab, may have a role in management.

Atypical hemolytic uremic syndrome (aHUS) or complement-mediated thrombotic microangiopathy occurs secondary to a derangement in complement resulting in dysregulated activity at the endothelial cell surface [12]. This activity results in microangiopathy and tissue function disruption, dependent on the location of cell damage. Eculizumab, a monoclonal antibody which inhibits complement pathway activation by inhibiting cleavage of C5, has been widely used and highly effective in the treatment of aHUS, yet its role in STEC-HUS is poorly defined [12–16].

In this article, we present a case of a 3-year-old boy with diarrhea-positive STEC-HUS with severe renal and neurologic involvement who showed improvement of symptoms after eculizumab administration.

## 2. Case Presentation

A 3-year-old male with no significant past medical history presented to the emergency department with a 3-day history of bloody diarrhea, vomiting, and poor oral intake. He had recently attended his grandmother's farm where he played with a chicken just prior to symptom onset.  Table 1 shows laboratory findings concerning for HUS, with thrombocytopenia, hemolytic anemia, schistocytes, elevated lactate dehydrogenase, and renal failure.

Stool culture from referring hospital was positive for Shiga-toxin 2 *E. coli.* Intravenous furosemide was attempted on day 1 of hospitalization despite which he remained anuric with stable electrolytes. Decision was made to proceed with intermittent hemodialysis (IHD).

**Figure 1:**
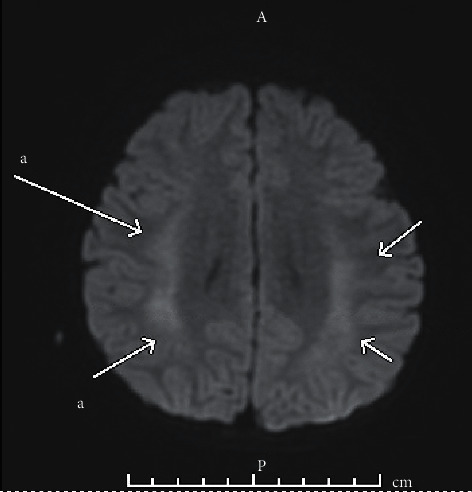
MRI findings on day 2 of hospitalization. Fluid-attenuated inversion recovery axial view shows bilateral frontoparietal lobe diffusion restriction, seen in acute encephalopathy or postictal changes.

On day 2 of hospitalization, after the second round of IHD, the patient developed focal tonic-clonic activity involving the left upper extremity and face, with eye fluttering, and remained postictal briefly with recovery of neurologic status shortly after. Intravenous lorazepam was administered with cessation of seizure activity. Immediate evaluation with MRI of brain showed nonspecific T2/FLAIR hyperintense signaling likely related to postictal changes or acute encephalopathy but no other acute abnormality (Figure 1). Magnetic resonance venography was normal.

On day 4 of hospitalization, the patient developed mild encephalopathy with delayed verbal/nonverbal responses and anisocoria. Computerized tomography of the brain was normal. An electroencephalogram showed diffuse slowing concerning for mild encephalopathy. He was started on intravenous levetiracetam for seizure prophylaxis. Given his clinical course and neurologic manifestations, the decision was made to initiate eculizumab therapy after a multispecialty meeting. He received prophylaxis with meningococcal conjugate vaccine (Menactra®), and labs for aHUS and thrombotic thrombocytopenic purpura (TTP) were sent prior to initiating an eculizumab dose of 600 mg followed by 300 mg/week. His anisocoria and encephalopathy gradually improved on day 5 of hospitalization. However, on day 7 of hospitalization, he developed decreased verbal response and interaction, along with more frequent intermittent twitching of his left lower extremity and left eye. Repeat magnetic resonance imaging was normal, and he was transitioned to continuous venovenous hemodiafiltration (CVVHDF) for more consistent management of azotemia. On day 8 of hospitalization, he developed right-sided hemineglect and continuous EEG captured four episodes of subclinical seizures emanating from the left hemisphere, but by day 9, he started to show daily gradual improvement in his neurological function, with complete resolution by day 21 of hospitalization.

**Figure 2:**
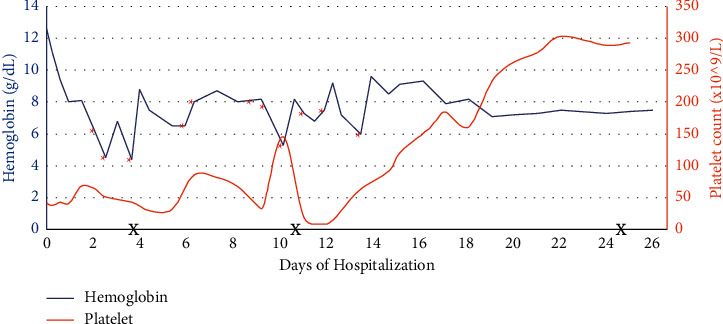
Trend of hemoglobin and platelets throughout the hospital course. Asterisks are reflective of red blood cell transfusions, and X is reflective of eculizumab administrations.

**Figure 3:**
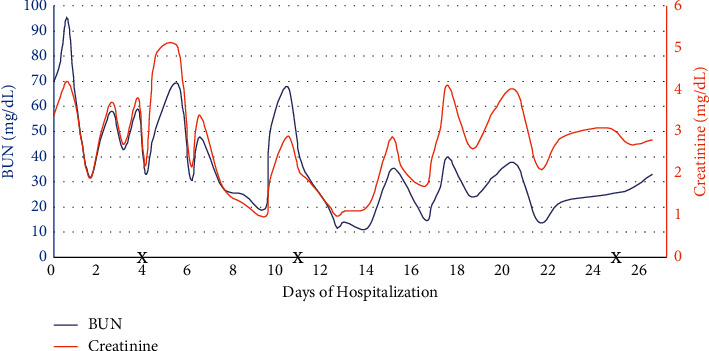
Trend of BUN and creatinine throughout the hospital course. X reflects eculizumab administration. Near daily sessions of hemodialysis occurred, with CRRT initiated days 7–17.

**Figure 4:**
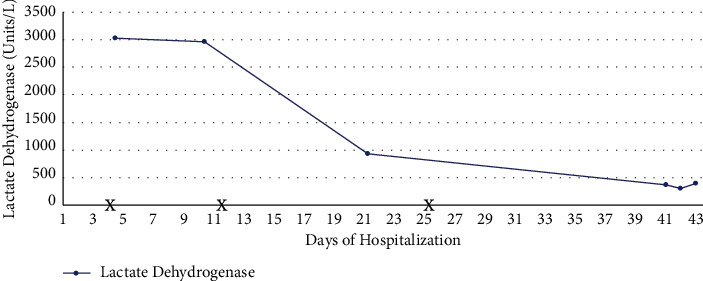
Trend of lactate dehydrogenase the throughout hospital course. X reflects eculizumab administration.

By day 21 of hospitalization, he had adequate urine output and renal replacement therapy was discontinued. At the time of discharge, his urine output had continued to improve and he showed no sign of residual neurological deficits. In total, he received 9 sessions of CVVHDF and 11 sessions of IHD. He also received 3 doses of eculizumab while inpatient. Trends of hemoglobin, platelets, BUN, creatinine, and lactate dehydrogenase are shown in Figures 2–4, respectively, which show improvement.

Outpatient follow-up revealed no subsequent seizures or encephalopathy. His urine output returned to normal, and furosemide was discontinued. At the final week (week 8) of eculizumab therapy, his labs showed resolution of HUS with a hemoglobin of 10.1 mg/dL, platelet count of 298 k/cumm, BUN 20 mg/dL, and creatinine of 1.2 mg/dl. Of note, his atypical HUS genetics panel was positive for mutations of unknown significance in genes ADAMTS13, CFHR4, MCP/CD46, and CFI but no known mutations that would classify him as atypical HUS. ADAMTS13 activity was initially low (25% with reference of 40–130%), but repeat was within normal limits at 46%. ADAMTS13 inhibitor activity was negative.

## 3. Discussion

This case demonstrates the systemic effects of STEC and how the central nervous system can be uniquely impacted in HUS, manifesting as seizure and encephalopathy. Direct renal endothelial damage is a well-understood pathophysiological principle of HUS that leads to the classic clinical features of acute kidney injury, thrombocytopenia, and hemolytic anemia. More recently, complement cascade activation and increased cellular response have emerged as important pathophysiological considerations in the disease progression [17]. Although there are no established guidelines for the treatment of STEC-HUS with focus on supportive care, successful implementation of immunoadsorption and eculizumab has reinforced the notion of the host immune response playing a role in the pathophysiology of STEC-HUS [11, 18–20].

With the increased utilization of monoclonal antibodies, eculizumab has been proposed as a potential treatment for STEC-HUS. Eculizumab has been approved for the treatment of aHUS, as it has been shown to significantly improve renal function in a time-dependent manner; however, it is increasingly being used in the treatment of STEC-HUS [20, 21]. Specifically, the use of eculizumab has been associated with the rapid amelioration of neurologic symptoms in several small cohorts [14, 22, 23]. It works by binding to complement protein C5, thus inhibiting the action of C5 convertase and, thereby, preventing the deployment of the terminal complement system, including the formation of the membrane attack complex (MAC) [24]. The role of complement is well documented in the pathogenesis of atypical HUS which involves inherited mutations in complement regulatory genes; however, the role of complement activation is less clear in STEC-HUS. In vitro assays have demonstrated biochemical evidence for the ability of Shiga toxin to activate the complement cascade, as well as bind factor H—an important negative regulator of the alternative complement pathway [25]. Due to loss of terminal complement activation, eculizumab has significantly elevated risk for developing meningococcal disease and infection from encapsulated bacteria (*S. pneumoniae* and *H. influenzae*) while undergoing therapy. To mitigate this risk, prophylactic meningococcal vaccination at least 2 weeks prior to treatment initiation is warranted. In cases where treatment cannot be delayed, vaccination should be initiated immediately. Antimicrobial prophylaxis with penicillin for the duration of therapy has also been recommended [26]. No formal treatment regimen has been established, but one clinical trial (ECULISHU) investigating eculizumab in the treatment of STEC-HUS mirrored the regimen of aHUS [27]. This included an induction dose dependent on the patient's weight and maintenance dosing 1 week after and then biweekly for a total of 8 weeks.

It is useful to acknowledge a possible continuum that may exist between typical STEC-HUS and aHUS. Although both diseases are rare, aHUS is due to intrinsic defects of the alternative complement pathway, while an infectious agent that when combined with atypical-causing mutations can potentially manifest a more severe disease state causes typical HUS. Thrombotic thrombocytopenic purpura (TTP) is another microangiopathic hemolytic disease that can have overlapping clinical features and similar pathophysiologic mechanisms as HUS [28–31]. TTP is secondary to a defect in ADAMTS13, which cleaves von Willebrand factor resulting in a similar presentation. Neurologic involvement in TTP is well documented, and eculizumab has also been used to treat refractory cases [32, 33]. Measurement of ADAMTS13 activity is key to differentiating TTP from other thrombotic microangiopathies and is dependent on an activity level under 10% [34]. ADAMTS13 level was low at 25%, but repeat testing was 46%, with neither meeting criteria for TTP. Furthermore, our patient's genetic screen returned positive for mutations within key complement regulatory proteins, notably, complement factor H (CFH), membrane cofactor protein (MCP), and complement factor I (CFI). The significance of these mutations is unknown; however, it supports the hypothesis that polymorphisms within complement regulatory genes may mitigate the disease course of typical HUS and provide rationale for a pharmacologic intervention such as eculizumab.

Important to note are other interventions such as therapeutic plasma exchange (TPE) that have been used in thrombotic microangiopathic diseases. TPE has been replaced by eculizumab as the first-line treatment for aHUS but remains the mainstay therapy for TTP [35]. The rationale to use TPE in severe cases of STEC-HUS includes the potential to decrease circulating cytokines, Shiga toxin, and “unusually large” von Willebrand factor multimers (ULVFWM) that contribute to proinflammatory and prothrombotic effects on the vascular endothelium. However, data are limited to support its routine use [36]. Decisions to include TPE specifically in management of neurologic involvement in STEC-HUS may be considered, but its efficacy is poorly defined and largely stems from adult cases of reported benefit in TTP [36]. Further studies investigating its effectiveness are warranted prior to routine implementation.

Although case reports similar to our own have shown resolution of acute neurological complications with eculizumab therapy, there exist studies that demonstrate no short-term benefit [11, 14, 22, 23, 37]. Given the complications of immunosuppression with eculizumab therapy and the unclear benefit in reducing long-term complications of HUS, clinical trials are warranted. To date, three separate clinical trials investigating the role of eculizumab in Shiga-toxin HUS exist (ECULISHU, NCT01410916, and ECUSTEC), but findings have not been published at this time [27, 38, 39]. These studies can potentially be instrumental in elucidating the utility and safety of eculizumab, in the management of severe sequelae from Shiga-toxin HUS in adults and children.

## 4. Conclusions

This case illustrated the clinical presentation and course of STEC-HUS with neurological complications that were ameliorated with eculizumab therapy. Diagnosis of STEC-HUS is made from stool culture identifying Shiga toxin in addition to findings of microangiopathic hemolytic anemia, thrombocytopenia, and symptoms of renal failure. Polymorphisms with complement regulatory genes exist with STEC-HUS and should be considered with severe neurologic and renal involvement. Randomized controlled trials are required to show benefits of eculizumab in such cases.

## Figures and Tables

**Table 1:** Initial laboratory findings consistent with hemolytic uremic syndrome.

WBC (k/cumm)	29.4
Hgb (g/dL)	12.6
Platelets (k/cumm)	42
LDH (*μ*/L)	3027
Haptoglobin (mg/dL)	16
BUN (mg/dL)	70
Creatinine (mg/dL)	3.4
Direct antiglobulin test	Negative
Sodium (mmol/L)	131
Potassium (mmol/L)	5.4
Chloride (mmol/L)	94
CO2 (mmol/L)	18
AST (*μ*/L)	339
ALT (*μ*/L)	148
Albumin (g/dL)	2.1
Indirect antiglobulin test (Coombs)	Negative

## Data Availability

Underlying data supporting the results of the study can be obtained by directly emailing the corresponding author of this article.
